# THC labeling on cannabis products: an experimental study of approaches for labeling THC servings on cannabis edibles

**DOI:** 10.1186/s42238-022-00124-1

**Published:** 2022-04-07

**Authors:** Samantha Goodman, David Hammond

**Affiliations:** School of Public Health Sciences, University of Waterloo, Waterloo, ON N2L 3G1 Canada

**Keywords:** Cannabis, Edibles, THC, Serving size, Labeling, Packaging

## Abstract

**Background:**

Over-consumption is a common adverse outcome from cannabis edibles. States such as Colorado require each serving of cannabis edible to carry a THC symbol. This study aimed to test whether packaging edibles in separate servings and/or indicating the THC level per serving improves consumer understanding of serving size.

**Methods:**

An 3 × 2 experimental task was conducted as part of the 2019 International Cannabis Policy Study online survey. Respondents from Canada and the US (*n* = 45,504) were randomly assigned to view an image of a chocolate cannabis edible. Packages displayed THC labels according to 1 of 6 experimental conditions: packaging (3 levels: whole multi-serving bar; individual chocolate squares; separately packaged squares) and THC stamp (2 levels: stamp on each square vs. no stamp). Logistic regression tested the effect of packaging and THC stamp on odds of correctly identifying a standard serving, among edible consumers and non-consumers separately. Edible consumers were also asked about their awareness of a standard THC serving.

**Results:**

Only 14.6% of edible consumers reported knowing the standard serving of THC for cannabis edibles. In the experimental task, among non-consumers who saw stamped bars, the multi-serving bar (AOR = 1.16 (1.08, 1.24) *p* < 0.001) and individually packaged squares (AOR = 1.08 (1.01, 1.16), *p* = 0.031) elicited more correct responses than individual squares. There was no difference in packaging formats when stamps were absent (*p* > 0.05 for all). Among edible consumers, there was no effect of the packaging (*p* = 0.992) or stamp manipulation (*p* = 0.988). Among both edible consumers and non-consumers, respondents in US states with legal recreational cannabis performed better than Canadians (*p* < 0.001).

**Conclusions:**

Regulations that require THC information to be stamped or indicated on each serving of cannabis edible may facilitate understanding of how much to consume, especially among novice consumers.

**Supplementary Information:**

The online version contains supplementary material available at 10.1186/s42238-022-00124-1.

## Introduction

Packaging and labeling are an important means for communicating product information to consumers, both for industry and regulatory authorities. Labeling practices are particularly important for communicating the ‘dose’ or potency of legal substances, including pharmaceutical products and “recreational” substances such as tobacco and alcohol (Kalsher et al. 1996; Stockwell et al. 2019; International Agency for Research on Cancer 2008). In jurisdictions that have legalized medical or non-medical cannabis, regulatory authorities have established requirements for health warnings, product contents, standardized cannabis symbols, and other measures to inform consumers (Kruger et al. 2021). To date, however, there is very little research on labeling standards for the wide diversity of cannabis products.

Labeling regulations for cannabis edibles warrant consideration for several reasons. Edibles account for an increasing proportion of the cannabis product market, with the edible industry expected to account for over USD $4.1 billion in Canada and the US by 2022 (Government of Canada 2019a; Barrus et al. 2016; The ArcView Group 2018). Despite the growing popularity of this product category, edibles present a risk in terms of accidental over-consumption. This is largely attributed to the delayed onset of psychoactive effects associated with oral ingestion compared to inhalation of THC, as well as high delta-9-tetrahydrocannabinol (THC) levels in some products (Barrus et al. 2016; Benjamin and Fossler 2016). Difficulties with edible dosing are exacerbated by the wide range of THC concentrations across different products: whereas some edibles contain several milligrams or even negligible levels of THC, others contain several hundred milligrams of THC (Mahamad et al. 2020). Although the side effects of over-consuming edibles are not fatal and typically short-term, they can be highly unpleasant and lead to the use of health care system resources (Vigil et al. 2018; Cao et al. 2016). To assist consumers with safe dose titration, US states that have legalized non-medical cannabis (“legal” states) have established “standard servings” of either 5 or 10 mg THC for cannabis edibles, although packages can contain up to 50 or 100 mg THC (Oregon Liquor Control Commission 2018; State of Massachusetts: Cannabis Control Commission 2019; Alaska Department of Commerce Community and Economic Development 2019; Colorado Department of Revenue: Marijuana Enforcement Division 2018; Washington State Legislature 2018; State of Nevada Department of Taxation 2017; California Department of Public Health 2019). Some jurisdictions have taken a more conservative approach. In Canada, where edibles became available for legal sale in early 2020, packages are permitted to contain a maximum of 10 mg THC (Government of Canada 2019b). Using a different approach, the State of Colorado mandates that each 10-mg THC serving carry the state’s universal THC symbol, and limits packages to 10 mg THC only in cases where labeling individual servings is infeasible (Colorado Department of Revenue: Marijuana Enforcement Division 2018).

To our knowledge, no studies have examined the efficacy of labeling individual servings of THC, and few studies have examined consumer understanding of THC information and serving size for cannabis edibles (Leos-Toro et al. 2020; Goodman and Hammond 2020). This study aimed to test whether packaging cannabis edibles in separate servings and indicating the THC level on each unit improves consumer understanding of serving size. It was expected that seeing chocolate packaged in individual units and/or with the THC level stamped on each unit would elicit superior understanding of serving size compared to other packaging formats and no THC stamp, respectively. A secondary objective was to examine consumer knowledge of standard servings of THC.

## Methods

Data are cross-sectional findings from the 2019 International Cannabis Policy Study (ICPS) online survey (Hammond et al. 2019), conducted in Canada and the US. Data were collected via self-completed web-based surveys in fall 2019 with respondents aged 16–65. Respondents were recruited through the Nielsen Consumer Insights Global Panel and their partners’ panels. Email invitations were sent to a random sample of panelists, after targeting for age and country criteria. Surveys were conducted in English in the US and English or French in Canada. Respondents provided consent prior to completing the survey and received remuneration in accordance with their panel’s usual incentive structure (e.g., points-based or monetary rewards, chances to win prizes). The study was reviewed by and received ethics clearance through a University of Waterloo Research Ethics Committee (ORE#31330). The full survey includes a comprehensive range of questions assessing cannabis consumption, purchasing behaviors, retail sources, and psychosocial risk factors. The current paper reports findings from an experimental study administered at the end of the ICPS survey, immediately after sections that assessed polysubstance use and opinions of cannabis policies. A full description of the study methods is published elsewhere (Hammond et al. 2020; Goodman et al. 2020a).

### Measures

#### Demographic information

Demographic information included sex, age, ethnicity, highest education level, and perceived income adequacy. See ICPS 2019 survey for item wording (Hammond et al. 2019) and Supplementary Table 1 for response options. Device type used to complete the survey was also collected and included in models to adjust for any effects of screen size when viewing images.

#### Respondent jurisdiction

Respondent jurisdiction was categorized according to legality of non-medical (recreational) cannabis as of August 2019: Canada (legal), US “legal” states, and US “illegal” states.^1^

#### Consumption of cannabis edibles

Respondents were asked, “Have you used marijuana in any of the following ways?” followed by a list of modes of administration. Past 12-month consumption of “Edibles/foods” was coded as a binary variable (Yes vs. No/Don’t know).

#### Knowledge of a standard serving of THC

To assess knowledge of THC serving size information, past 12-month edible consumers were asked, “In places where marijuana is legal, governments use a standard serving of THC. Do you know what the standard serving is [where you live?]” (Yes, No, Don’t know, Refuse). If yes: “How much THC is in one standard serving of an edible [where you live?]”.^2^ Because no jurisdiction has implemented a standard serving greater than 10 mg THC, values of 1–10 mg THC were considered plausible.

#### Experimental task

**Fig. 1:**
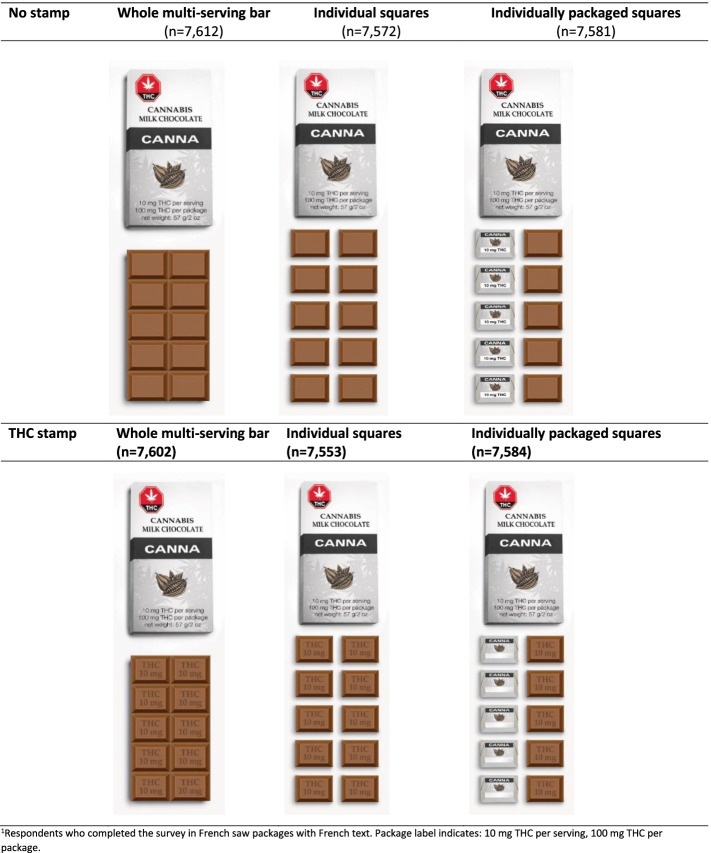
THC labeling images on cannabis edible packaging by study condition (*n* = 45,504). Respondents who completed the survey in French saw packages with French text. Package label indicates: 10 mg THC per serving, 100 mg THC per package

Upon conclusion of the main ICPS survey, respondents were shown an image of a cannabis chocolate bar on the screen. Images were displayed according to 1 of 6 randomly assigned conditions, as shown in Fig. 1. A 3 × 2-experimental design was used in which two factors were manipulated: packaging condition (3 levels: whole multi-serving chocolate bar; individual squares; individually packaged squares) and THC stamp (2 levels: THC level stamped on each square vs. no stamp). All packages indicated 10 mg THC per serving and 100 mg THC per package. While viewing the image, respondents were asked, “How many squares of chocolate should someone eat if they want one serving of THC?” (Less than 1 square, 1,2, 3, 4, 5, 6, 7, 8, 9, 10, More than 10, Don’t know, Refuse). To account for jurisdictional differences in serving size (5 vs. 10 mg), responses of “Less than 1 square” or “1 square” were coded as correct.

### Data analysis

The 2019 ICPS cross-sectional sample comprised 45,735 respondents; the current sample consisted of 45,504 respondents after excluding 231 who refused to answer the experimental question. Binary logistic regression was used to test the effects of packaging format and presence of THC stamp on odds of responding correctly to the experimental task (Correct vs. Incorrect/Don’t know). The two-way interaction between the packaging and stamp manipulations was tested in a subsequent model. Models were stratified by cannabis consumption in the past 12 months: (1) no cannabis consumption, (2) “any” cannabis consumption, and (3) consumption of cannabis edibles in the past 12 months. Adjusted odds ratios (AORs) and 95% confidence intervals are shown. All odds ratios are adjusted for age, sex, jurisdiction, ethnicity, education, perceived income adequacy and survey device type. Chi-squared tests examined differences between (a) experimental conditions in distribution of the aforementioned covariates and (b) jurisdictions in reported knowledge of standard serving of THC. Analyses were conducted using SAS Studio 9.4.

## Results

See Supplementary Table 1 for sample characteristics. Mean respondent age was 41.9 (SD = 14.4) years, 69.7% were female, and the majority (79.3%) had at least some college/university education. There were no significant differences in the distribution of covariates across experimental conditions (*p* > 0.05 for all; see Supplementary Table 1).

### Knowledge of standard servings of THC

Overall, only 14.6% (*n* = 1,196) of past 12-month edible consumers reported knowing the standard serving of THC; the majority (85.4%; *n* = 7,018) stated “No” or “Don’t know”. Reported knowledge differed by jurisdiction (*X*^*2*^(2) = 63.61, *p* < 0.001) and was highest in US “legal” states (17.1%) followed by US “illegal” states (13.5%) and Canada (9.9%).

Of those who reported knowing the standard serving, 845 respondents entered a THC value. The average value (mean = 26.7 mg, SD = 95.6 mg) was similar across jurisdictions (Canada = 27.3 mg, US “illegal” states = 29.9 mg, US “legal” states = 25.8 mg). The mode and median in all jurisdictions were 10 mg THC. The majority of respondents (72.3%; *n* = 611) indicated a plausible value (i.e., 1–10 mg THC)^3^, including 66.7% (*n* = 88) in Canada, 67.2% (*n* = 86) in US “illegal” states, and 74.4% (*n* = 437) in US “legal” states, with no difference between jurisdictions (*p* = 0.085). Regardless of what value was reported, those who reported knowing the standard serving of THC were *less* likely to respond correctly to the experimental task compared to those who reported not knowing (12.8% vs. 87.2%, *X*^*2*^(1) = 31.25, *p* < 0.001).

### Results of experimental task

**Fig. 2:**
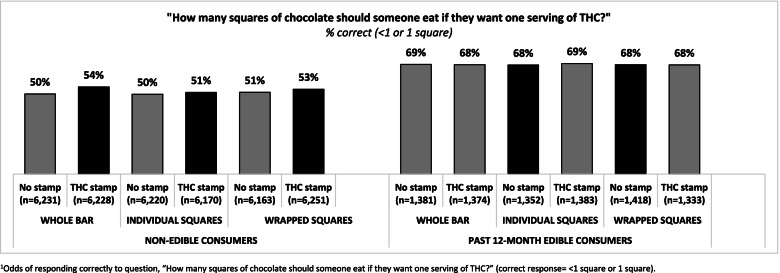
Accuracy in identifying THC servings displayed on cannabis edible packaging by experimental question (*n* = 45,504). Odds of responding correctly to question, “How many squares of chocolate should someone eat if they want one serving of THC?” (correct response = < 1 square or 1 square)

When shown the chocolate bar image, approximately half of the sample in any condition correctly reported that an individual should eat “Less than 1 square” or “1 square” to consume one serving of THC. As shown in Fig. 2, stratifying results by history of edible consumption revealed that approximately half of non-consumers in any condition responded correctly (range = 48.5–52.7%), compared to approximately two thirds of any past 12-month cannabis consumers (range = 62.3–65.0%). This difference was largely driven by respondents who selected “Don’t know” (coded as incorrect): 36.6% of non-consumers versus 13.3% of consumers indicated not knowing the standard serving (see Supplementary Fig. 1).

Table 1 shows the results of the logistic regression model testing the odds of a correct response on the experimental task, stratified by cannabis consumption in the past 12 months. Among non-cannabis consumers, the effects of packaging condition (*p* = .004) and stamp manipulation (*p* < .001) were associated with greater accuracy of judging THC content. Among respondents who had consumed “any” cannabis product in the past 12 months, only the stamp manipulation was significant (*p* = .028), whereas there was no effect of either packaging or stamp conditions among cannabis edible consumers—see Table 1.

**Table 1:** Accuracy of judging THC content based on “edible” cannabis packaging and consumer status (*n* = 45,504)

Variable	No cannabis consumption in past 12 months (*n* = 29,731)		“Any” cannabis consumption in past 12 months (*n* = 15,773)		Edible cannabis consumption in past 12 months (*n* = 8241)	
	AOR (95% CI)	***p***-value	AOR (95% CI)	***p***-value	AOR (95% CI)	***p***-value
**Packaging manipulation**	***X***^***2***^**(2) = 10.86**	**0.004**	*X*^2^(2) = 0.08	0.959	*X*^*2*^(2) = 0.02	0.992
Multi-serving bar vs. individual squares	**1.09 (1.03, 1.16)**	**0.002**	1.01 (0.93, 1.10)	0.791	0.99 (0.88, 1.12)	0.913
Individually packaged squares vs. individual squares	**1.08 (1.02, 1.14)**	**0.010**	1.00 (0.92, 1.09)	0.972	1.00 (0.89, 1.12)	0.995
Individually packaged squares vs. multi-serving bar	0.99 (0.93, 1.04)	0.615	0.99 (0.91, 1.07)	0.817	1.01 (0.90, 1.13)	0.907
**Stamp manipulation**	***X***^***2***^**(1) = 15.13**	**< 0.001**	***X***^***2***^**(1) = 4.80**	**0.028**	*X*^*2*^(2) = 0.00	0.988
No stamp *(ref)*	--ref--	--ref--	--ref--	--ref--	--ref--	--ref--
THC stamp	**1.10 (1.05, 1.15)**	**< 0.001**	**1.08 (1.01, 1.15)**	**0.028**	1.00 (0.91, 1.10)	0.988
**Age group**	***X***^***2***^**(4) = 447.77**	**< 0.001**	***X***^***2***^**(4) = 54.80**	**< 0.001**	***X***^***2***^**(4) = 10.41**	**0.034**
16–25 *(ref)*	--ref--	--ref--	--ref--	--ref--	--ref--	--ref--
26–35	**0.86 (0.79, 0.94)**	**< 0.001**	0.95 (0.86-1.06)	0.339	1.02 (0.88, 1.17)	0.839
36–45	**0.77 (0.71, 0.84)**	**< 0.001**	**0.88 (0.79, 0.98)**	**0.022**	0.97 (0.83, 1.13)	0.680
46–55	**0.64 (0.58, 0.69)**	**< 0.001**	**0.83 (0.73, 0.93)**	**0.001**	0.89 (0.75, 1.05)	0.161
56–65	**0.47 (0.43, 0.51)**	**< 0.001**	**0.68 (0.60, 0.76)**	**< 0.001**	**0.81 (0.68, 0.96)**	**0.014**
**Sex**	***X***^***2***^**(1) = 91.15**	**< 0.001**	***X***^***2***^**(1) = 122.45**	**< 0.001**	***X***^***2***^**(1) = 78.78**	**< 0.001**
Female *(ref)*	--ref--	--ref--	--ref--	--ref--	--ref--	--ref--
Male	**0.78 (0.74, 0.82)**	**< 0.001**	**0.67 (0.62, 0.72)**	**< 0.001**	**0.63 (0.57, 0.70)**	**< 0.001**
**Jurisdiction**	***X***^***2***^**(1) = 167.31**	**< 0.001**	***X***^***2***^**(2) = 96.68**	**< 0.001**	***X***^***2***^**(2) = 62.11**	**< 0.001**
Canada *(ref)*	--ref--	--ref--	--ref--	--ref--	--ref--	--ref--
US “illegal” states	**1.28 (1.21, 1.37)**	**< 0.001**	0.98 (0.89, 1.08)	0.710	0.93 (0.81, 1.07)	0.304
US “legal” states	**1.43 (1.35, 1.51)**	**< 0.001**	**1.39 (1.29, 1.50)**	**< 0.001**	**1.42 (1.27, 1.59)**	**< 0.001**
**Education**	***X***^***2***^**(4) = 48.78**	**< 0.001**	***X***^***2***^**(1) = 51.70**	**< 0.001**	***X***^***2***^**(4) = 42.37**	**< 0.001**
Less than high school *(ref)*	--ref--	--ref--	--ref--	--ref--	--ref--	--ref--
High school diploma or equivalent	**0.89 (0.80, 0.99)**	**0.032**	0.92 (0.80, 1.06)	0.270	0.96 (0.77, 1.19)	0.701
Some college/university or technical training	1.06 (0.95, 1.17)	0.287	**1.20 (1.05, 1.38)**	**0.008**	**1.32 (1.07, 1.62)**	**0.009**
Bachelor’s degree or higher	1.05 (0.94, 1.17)	0.372	1.07 (0.93, 1.24)	0.336	1.13 (0.91, 1.41)	0.256
Unstated	**0.44 (0.31, 0.64)**	**< 0.001**	**0.35 (0.20, 0.62)**	**< 0.001**	**0.18 (0.07, 0.47)**	**< 0.001**
**Ethnicity**	***X***^***2***^**(1) = 112.42**	**< 0.001**	***X***^***2***^**(1) = 78.42**	**< 0.001**	***X***^***2***^**(1) = 46.71**	**< 0.001**
White *(ref)*	--ref--	--ref--	--ref--	--ref--	--ref--	--ref--
Other/mixed/unstated	**0.73 (0.69, 0.78)**	**< 0.001**	0.70 (0.64, 0.76)	**< 0.001**	**0.68 (0.61, 0.76)**	**< 0.001**
**Perceived income adequacy (difficulty making ends meet)**	***X***^***2***^**(5) = 184.80**	**< 0.001**	***X***^***2***^**(5) = 79.67**	**< 0.001**	***X***^***2***^**(5) = 36.25**	**< 0.001**
Very difficult *(ref)*	--ref--	--ref--	--ref--	--ref--	--ref--	--ref--
Difficult	0.91 (0.84, 1.00)	0.051	1.05 (0.93, 1.18)	0.452	1.08 (0.91, 1.29)	0.370
Neither easy nor difficult	**0.78 (0.72, 0.85)**	**< 0.001**	0.94 (0.84, 1.06)	0.302	0.94 (0.79, 1.11)	0.446
Easy	**0.85 (0.77, 0.93)**	**< 0.001**	0.94 (0.83, 1.07)	0.376	1.00 (0.83, 1.21)	0.978
Very easy	**0.72 (0.65, 0.80)**	**< 0.001**	**0.75 (0.65, 0.86)**	**< 0.001**	**0.95 (0.77, 1.17)**	**0.017**
Unstated	**0.36 (0.31, 0.43)**	**< 0.001**	**0.42 (0.33, 0.54)**	**< 0.001**	**0.45 (0.31, 0.64)**	**< 0.001**

Odds of responding correctly to question, “How many squares of chocolate should someone eat if they want one serving of THC?” (correct response = < 1 square or 1 square). Logistic regression models, adjusting for age sex, education, ethnicity, income adequacy, and survey device type. Each column shows results stratified by subsample based on cannabis consumption status

“Legal” states refer to US states in which non-medical cannabis was legal at the time the study was completed

*AOR* Adjusted odds ratio, *ref* Reference category, *SD* Standard deviation, *95% CI* 95% confidence interval, *X*^*2*^*(df)* Chi-squared statistic (degrees of freedom)

A subsequent model among non-cannabis consumers in the past 12 months revealed a significant two-way interaction between the packaging and stamp manipulations (*X*^2^(2) = 8.41, *p* = 0.015). The model indicated that, among non-consumers who saw stamped bars, the odds of responding correctly were higher when respondents saw either a multi-serving bar (AOR = 1.20 (1.11, 1.30) *p* < 0.001) or individually packaged squares (AOR = 1.08 (1.00, 1.18), *p* = 0.049) compared to individual squares. There was no difference between individually packaged squares versus the multi-serving bar when stamps were present, nor was there an effect of packaging among those who saw unstamped bars (*p* > 0.05 for all contrasts). There was no significant two-way interaction between the packaging and stamp manipulations in a model among consumers who had consumed any cannabis in the past 12 months (*X* ^2^(2) = 1.40, *p* = 0.497).

Table 1 also shows the effects of socio-demographic covariates in the main effects models. Briefly, the following groups were significantly more likely to respond correctly: older adults (vs. 16–25-year-olds), females (vs. males), less than high school education (vs. unstated), White respondents (vs. other/mixed/unstated ethnicity), and those with the lowest income adequacy (vs. unstated and certain higher levels of income adequacy). In addition, compared to Canada, non-consumers residing in all US jurisdictions were significantly more likely to respond correctly; among consumers, this was only true among those residing in US “legal” states.

## Discussion

Overall, very few (< 15%) cannabis edible consumers reported knowing the standard serving of THC. This low reported knowledge is consistent with previous research from our group in which less than 15% of those in “illegal” jurisdictions and under a quarter in US “legal” states were able to report the THC level of the cannabis edibles they usually consumed (Hammond and Goodman 2020). Based on standard servings of 5 and 10 mg in jurisdictions with legal cannabis sales, the average value provided by consumers (26.7 mg) was equivalent to approximately 2.5 to 5 servings of THC. More promisingly, three quarters of edible consumers in US “legal” states provided a plausible THC value (i.e., 10 mg or less), compared to only two-thirds of respondents in US “illegal” states—who would expected to be less familiar with labeled THC values—and Canada, where legal edibles only became available for sale in early 2020, after the current study period (Government of Canada 2019b, d). If jurisdictions reach a consensus on a “standard serving” of THC (Freeman and Lorenzetti 2019; Hammond 2021; National Institute on Drug Abuse (NIDA) 2021), we expect familiarity with this term to increase among consumers.

In the experimental task, understanding of serving sizes for cannabis edibles was poor among non-cannabis consumers, with only less than half selecting the correct response of 1 square (10 mg THC) or less than 1 square based on information provided on the package. This generally poor understanding of THC labeling and serving size is consistent with previous studies on consumer understanding of serving sizes for cannabis edibles (Leos-Toro et al. 2020; Goodman and Hammond 2020; Hammond and Goodman 2020). In contrast, over two thirds of edible consumers were able to correctly select the serving size when presented with the information on the package. This finding is logical: those who have previously consumed edibles and/or seen THC labeling on edibles would be expected to better understand THC serving size information. This may reflect the overall low level of knowledge of standard servings and THC information among those who do not routinely consume edibles or may reflect disengagement with the survey question due to a disinterest in consuming edibles. When considering only those who actually selected a serving size, results were much more comparable between consumers and non-consumers.

Findings from the experimental manipulation of individually packaged servings and THC “stamps” suggest that these measures enhanced the accuracy of THC estimates, but only for non-consumers. When non-consumers were shown individually packaged servings stamped with the THC level, they better understood serving size compared to when they were shown individual unpackaged servings. Contrary to expectations, this improved performance was also observed for the multi-serving bar compared to individual unpackaged servings. The reason for the latter finding is unclear, although it is plausible that when respondents saw a multi-serving bar, the THC stamps were more salient and helped respondents understand how much to consume. In previous research, packaging individual doses of a cannabis edible led to better understanding of serving size compared to a multi-serving edible (Goodman and Hammond 2020). However, given that even the multi-serving bar in the current study had discernible servings (i.e., demarcated squares) and considering the effectiveness of unit-dose packaging in the previous study (Goodman and Hammond 2020), the most effect labeling approach would be to combine and extend the approaches taken in Canada and Colorado by restricting the amount of THC per container *and* clearly demarcating and labeling each serving of THC.

Differences in understanding were also observed between socio-demographic subgroups. Consistent with previous research on cannabis edibles, improved understanding was observed among females, younger respondents, majority ethnic groups, and states with legal recreational cannabis (Goodman and Hammond 2020). The superior performance among females is consistent with greater use of nutrition labels on foods as well as greater consumption of edibles and oral forms of cannabis among females compared to males (Government of Canada 2019a; Campos et al. 2011; Cuttler et al. 2016). The low levels of understanding among older respondents and respondents who did not identify as “white” highlight a potential need for targeted educational efforts. Those in US “legal” states are more likely to have been exposed to legal cannabis packaging with THC information, which may explain their higher level of understanding. Consumption of cannabis edibles has also been shown to be higher in US “legal” states (Goodman et al. 2020b), many of which had more mature retail cannabis markets compared to Canada at the time of the study. The findings are broadly similar to studies examining awareness of health warnings, which are also higher in states that have legalized non-medical cannabis and require mandated warnings and other packaging standards (Goodman and Hammond 2021).

Finally, in line with previous research, those who reported knowing the standard serving of THC performed more poorly when asked to identify a standard serving in the experimental task (Goodman and Hammond 2020). It seems that perceived knowledge of THC information may not be representative of actual understanding. It is also plausible that respondents who reported knowing the standard serving of THC did not read the label when shown the chocolate bar image. However, the experimental task was placed much later in the survey compared to the knowledge question, making it unlikely that the question regarding standard servings of THC influenced results on the experimental task, and there was no association between the two measures.

### Limitations

Respondents were recruited using non-probability-based sampling; therefore, the findings do not provide nationally representative estimates. Although response bias is a common limitation in survey research (and perhaps especially in US jurisdictions were cannabis use is prohibited), respondents were asked, “Were you able to provide ‘honest’ answers about your marijuana use during the survey?” and were excluded from the larger sample if they responded “no” (*n* = 717; 1.4%). Respondents may have not been familiar with the term “standard serving” for THC or may have misinterpreted the question to be asking about the serving size they typically consume. The experimental task was presented near the end of an online survey using embedded images. Some respondents may have had difficulty reading the serving size information, may not have scrutinized the information on the package, and/or may have been less engaged with the question than would be expected in a stand-alone study or naturalistic setting. Thus, although packages were designed to mimic those available on the online market, it is likely that the online experimental design underestimated the impact of packaging and labeling cannabis edibles. This is especially likely to be the case for packaging standards that require products to be packaged in separate units or “pieces,” which would be much more noticeable and concrete in the context of actual product packaging than is the case for images presented to consumers in an online study.

## Conclusions

Very few consumers were aware of the standard serving size for THC edibles. Indicating the THC level on each serving of a cannabis edible may improve understanding of how much to consume, especially among novice consumers. The magnitude of effect of the labeling measures examined in the current study was modest; however, the impact of similar regulations could have a considerable population-level impact under real-world conditions. Future studies should examine packaging standards that require “unit-dose” packaging in greater detail. In particular, Canada currently requires that edibles are sold in packages that contain no more than 10 mg of THC, regardless of the number of individual units or pieces. Studies should assess the extent to which these regulations enhance consumer understanding of THC and reduce the likelihood of consumption in legal cannabis markets.

## Supplementary Information

**Additional file 1: Supplemental Table 1.** Sample characteristics and sample distribution across experimental conditions (*n* = 45,504).

**Additional file 2: Supplementary Figure 1.** Response options among edible consumers and non-consumers (*n* = 45,504).

## Data Availability

The datasets used and/or analyzed during the current study are available from the corresponding author on reasonable request.

## Abbreviations

AOR: Adjusted odds ratio
ICPS: International Cannabis Policy Study
THC: Delta-9-tetrahydrocannabinol
US: United States

## Footnotes

1 ‘Legal’ states as of August 2019: Alaska, California, Colorado, Maine, Massachusetts, Michigan, Nevada, Oregon, Vermont, Washington, and District of Columbia. The remaining US states were considered “illegal.”

2 For both questions, the phrase “where you live” was omitted for those who resided in US “illegal” states.

3 Values of up to 10 mg were considered plausible, given that legal US and Canadian jurisdictions use 5 mg or 10 mg as the standard serving of THC, and regulators have suggested that consumers start with lower values (e.g., 2.5 mg THC) (Government of Canada 2019c).
