# Supplementary material for: THC labeling on cannabis products: an experimental study of approaches for labeling THC servings on cannabis edibles
Source: J Cannabis Res. 2022 Apr 7;4:17. doi: 10.1186/s42238-022-00124-1 (PMC8988394; doi:10.1186/s42238-022-00124-1)
Supplement: Supplementary file 1 — Additional file 1: Supplemental Table 1. Sample characteristics and sample distribution across experimental conditions (n = 45,504). [file 42238_2022_124_MOESM1_ESM.docx]

Supplemental Table 1. Sample characteristics and sample distribution across experimental conditions (n=45,504)

| **Variable** | **Non-consumers** | **Past 12-month edible consumers** | **Distribution of covariates between consumers and non-consumers** | **Distribution of covariates across six experimental conditions** |
| --- | --- | --- | --- | --- |
|  | **% (n)** | **% (n)** | ***X*^2^(df), p-value** | ***X*^2^(df), p-value** |
| **Age group** |  |  | **836.02(4), p<0.001** | 18.93(20), p=0.526 |
| 16-25 | 15.2% (5,667) | 19.0% (1,563) |  |  |
| 26-35 | 19.5% (7,274) | 29.6% (2,437) |  |  |
| 36-45 | 19.4% (7,233) | 21.6% (1,777) |  |  |
| 46-55 | 19.5% (7,283) | 15.1% (1,245) |  |  |
| 56-65 | 26.3% (9,806) | 14.8% (1,219) |  |  |
| **Sex** |  |  | 0.01(1), p=0.910 | 2.25(5), p=0.814 |
| Female | 69.7% | 69.8% |  |  |
| Male | 30.3% | 30.2% |  |  |
| **Jurisdiction** | 34.9% | 26.4% | **598.80(2), p<0.001** | 6.26(10), p=0.793 |
| Canada (legal) | 23.6% | 17.3% |  |  |
| US ‘illegal’ states | 41.5% | 56.3% |  |  |
| US ‘legal’ states |  |  |  |  |
| **Education level** |  |  | **202.19(4), p<0.001** | 23.14(20), p=0.282 |
| Unstated | 0.6% (213) | 0.4% (36) |  |  |
| Less than high school | 7.2% (2,663) | 6.1% (500) |  |  |
| High school diploma or equivalent | 18.3% (6,828) | 19.9% (1,641) |  |  |
| Some college/university or technical training | 38.5% (14,351) | 45.3% (3,733) |  |  |
| Bachelor’s degree or higher | 35.5% (13,208) | 28.3% (2,331) |  |  |
| **Ethnicity** |  |  | 0.26(1), p=0.612 | 4.63(5), p=0.463 |
| White | 77.7% (28,935) | 77.4% (6,378) |  |  |
| Other/mixed/unstated | 22.4% (8,328) | 22.6% (1,863) |  |  |
| **Perceived income adequacy (difficulty making ends meet)** |  |  | **65.05(5), p<0.001** | 22.92(25), p=0.577 |
| Unstated | 3.0% (1,106) | 2.0% (164) |  |  |
| Very difficult | 10.1% (3,775) | 11.3% (929) |  |  |
| Difficult | 22.9% (8,523) | 25.6% (2,113) |  |  |
| Neither easy nor difficult | 33.7% (12,555) | 32.8% (2,704) |  |  |
| Easy | 19.9% (7,395) | 18.7% (1,538) |  |  |
| Very easy | 10.5% (3,909) | 9.6% (793) |  |  |
| **Survey device** |  |  | **280.87(2), p<0.001** | 7.85(10), p=0.644 |
| Smartphone | 48.6% (18,119) | 58.6% (4,830) |  |  |
| Tablet | 8.3% (3,087) | 5.6% (465) |  |  |
| Computer | 43.1% (16,057) | 35.8% (2,946) |  |  |

*X*^2^,chi-squared statistic; df, degrees of freedom.
