# Supplementary material for: THC labeling on cannabis products: an experimental study of approaches for labeling THC servings on cannabis edibles
Source: J Cannabis Res. 2022 Apr 7;4:17. doi: 10.1186/s42238-022-00124-1 (PMC8988394; doi:10.1186/s42238-022-00124-1)
Supplement: Supplementary file 2 — Additional file 2: Supplementary Figure 1. Response options among edible consumers and non-consumers (n = 45,504). [file 42238_2022_124_MOESM2_ESM.docx]

**Supplementary Figure 1. Response options among edible consumers and non-consumers** **(n=45,504)**
